# Reaction phenotypes in IgE-mediated food allergy and anaphylaxis

**DOI:** 10.1016/j.anai.2019.12.023

**Published:** 2020-05

**Authors:** Kok Wee Chong, Monica Ruiz-Garcia, Nandinee Patel, Robert J. Boyle, Paul J. Turner

**Affiliations:** ∗ Section of Inflammation, Repair, and Development, National Heart and Lung Institute, Imperial College London, London, United Kingdom; † Allergy Service, Department of Paediatric Medicine, KK Women’s and Children’s Hospital, Singapore; ‡ Discipline of Paediatrics and Child Health, School of Medicine, University of Sydney, Sydney, Australia

## Abstract

**Objective:**

Food allergy encompasses a range of food hypersensitivities. Different clinical phenotypes for food allergy likely exist in much the same way as endotype discovery is now a major research theme in asthma. We discuss the emerging evidence for different reaction phenotypes (ie, symptoms experienced after allergen exposure in food allergic individuals) and their relevance for clinical practice.

**Data Sources:**

Published and unpublished literature relating to reaction phenotypes in food allergy.

**Study Selections:**

Authors assessment of the available data.

**Results:**

Food anaphylaxis may be pathophysiologically different than anaphylaxis caused by nonfood triggers. Currently, there are no robust, clinically useful predictors of severity in food allergy. It is likely that patient-specific reaction phenotypes exist in food allergy, which may affect the risk of severe anaphylaxis. Allergen immunotherapy may modulate these phenotypes.

**Conclusion:**

Data are emerging to confirm our clinical experience that many food allergic patients experience stereotypical symptoms after allergen exposure, both in the community and at supervised oral food challenge, in a manner that varies among patients. Integrating data sets from different cohorts and applying unbiased machine-based learning analyses may demonstrate specific food allergy endotypes in a similar way to asthma. Whether this results in improvements in patient management (eg, through facilitating risk stratification or affecting the decision to prescribe an epinephrine autoinjector and, perhaps, the number of devices) remains to be determined, but given our current inability to predict which patients are most at risk of severe food allergic reactions, this will clearly be an important area of research in the future.

**Key Messages**

• Food anaphylaxis may be pathophysiologically different than anaphylaxis caused by nonfood triggers.
• Currently, there are no robust, clinically useful predictors of severity in food allergy.
• It is likely that patient-specific reaction phenotypes exist in food allergy, which may affect the risk of severe anaphylaxis.
• Allergen immunotherapy may modulate these phenotypes.
• Machine-based learning may help with endotype discovery in anaphylaxis.

## Introduction

Food allergy encompasses a range of food hypersensitivities. Different clinical phenotypes for food allergy are likely to exist in much the same way as endotype discovery is now a major research theme in asthma. In this review, we discuss the emerging evidence for different reaction phenotypes (ie, symptoms experienced after allergen exposure in food allergic individuals) and their relevance for clinical practice.

## Primary IgE-Mediated Food Allergy vs Pollen Food Allergy Syndrome

Primary food allergy results from primary sensitization to a food allergen. In contrast, secondary food allergy, also known as pollen food allergy syndrome (PFAS), refers to where the primary sensitization is to aeroallergens, with symptoms occurring because of exposure to cross-reactive allergens in food. Patients with PFAS experience oropharyngeal symptoms of itching or tingling with or without mild lip swelling after ingestion of specific fresh fruit or vegetable.^1^ The term *oral allergy syndrome* (OAS) is often used interchangeably with PFAS, but is a more general term describing *any* oropharyngeal symptoms that occur as part of an allergic reaction^2^ and will often progress to systemic symptoms, including anaphylaxis (ie, OAS occurs in both PFAS and primary food allergy). Indeed, more than 50% of patients described by Amlot et al^2^ in the first case series of OAS later experienced systemic symptoms and anaphylaxis.

Drawing a distinction between primary and secondary food allergy is vital in both risk stratification and management advice^1^ but may be tricky because patients will often have a mix of both primary food allergy and PFAS to different allergens, whereas others may even demonstrate a pattern of sensitization consistent with primary allergy and PFAS to the same allergen (eg, IgE positivity to both Ara h 2 and Ara h 8 for peanut or Cor a 1 and Cor a 8 for hazelnut). PFAS is usually considered to be a relatively benign condition in which systemic symptoms are rare because of lability of the causative allergens, such as Bet v 1 homologues and profilins.^3^ However, approximately 10% of PFAS presentations are associated with systemic symptoms and 1% to 2% with anaphylaxis.^4^ It is not clear why some patients experience more significant symptoms and whether this is due to polysensitization to more stable allergens, such as lipid transfer proteins^5^ or diagnostic misclassification.^6^^,^^7^ Component resolved diagnostics have improved our ability to discriminate between PFAS and primary food allergy but are only of limited use in identifying patients with PFAS at risk of severe systemic reactions.^8^^,^^9^ Whether prescription of epinephrine autoinjector devices is indicated in patients with PFAS remains unclear.^4^^,^^6^^,^^7^

## Is Food-Induced Anaphylaxis: Pathophysiologically Different Than Nonfood Anaphylaxis?

Anaphylaxis is defined as “a serious systemic hypersensitivity reaction that is usually rapid in onset and may cause death. Severe anaphylaxis is characterized by potentially life-threatening compromise in breathing and/or the circulation, and may occur without typical skin features or circulatory shock being present.”^10^ Distinct differences appear to exist in the epidemiology and clinical presentations of anaphylaxis caused by food compared with other triggers, such as medication or insect venom (Table 1).^11^ These differences raise key questions about the underlying mechanisms involved. Food-related anaphylaxis (as defined according to the latest World Allergy Organization criteria^10^) tends to result in predominantly respiratory symptoms (with or without other organ involvement); cardiovascular compromise tends to be less common and, if present, occurs in the context of severe respiratory symptoms; the most common mode of death in fatal food anaphylaxis is respiratory arrest^12^^,^^13^; and when cardiovascular arrest occurs in patients with food anaphylaxis, it is usually secondary to respiratory compromise.^11^

**Table 1:** Epidemiologic and Pathphysiologic Differences of Anaphylaxis Due to Food vs Nonfood Causes^a^

Variable	Food	Medication or iatrogenic causes	Venom sting
Age distribution for anaphylaxis	Most common in preschool children, less common in older adults	Predominantly older ages	All ages, but less common in children
Age distribution for fatal reactions	Adolescents and young adults; rare in younger children and older adults	Older adults and elderly individuals	Middle-aged or older adults
Symptoms	Predominantly respiratory	Cardiovascular (respiratory less common)	Cardiovascular (respiratory less common)
Asthma or atopy	Common	Uncommon	Uncommon
Reaction onset	Typically within 2 hours of ingestion	More rapid	More rapid
Route of antigen presentation	Usually orogastric route	Usually parenteral	Parenteral
Mast cell tryptase	Usually no or only a relatively modest increase observed	Usually increases	Usually increases
Sex	Prevalence similar in males and females	Prevalence similar in males and females	More frequent in males than females

a Adapted from Turner and Campbell.^11^

These differences could, perhaps, be explained by the different routes of exposure (ie, food allergens need to be absorbed through the gastrointestinal tract, resulting in slower onset of symptoms and slower systemic absorption) compared with parenteral allergens. Furthermore, food processing can affect the kinetics of any ensuing reaction (eg, many patients with allergy to egg in a baked food matrix (such as a cake) present with more delayed (and typically abdominal) symptoms^14^^,^^15^ than when challenged to the native allergen, which might reflect the more complex food presentation, resulting in delayed absorption. Interestingly, modifying the presentation of the allergen through baking does not appear to change the sensitivity or reaction threshold (ie, amount of allergen needed to trigger an objective reaction), just the nature of the resulting symptoms.^16^

Despite this, there is evidence that food allergens can be rapidly absorbed through the buccal mucosa. Dirks et al^17^ described 6 nonallergic individuals who chewed peanut for 2 minutes after which they expelled the peanut without swallowing; under these circumstances, chewing alone resulted in allergen levels in the circulating blood that were sufficient to trigger an effector cell response.^17^ Furthermore, oral medications (eg, antibiotics) frequently result in cardiovascular symptoms despite the need to undergo gastrointestinal absorption.^12^ Therefore, the route of exposure does not explain these differences.

Alternatively, the association between food anaphylaxis and respiratory compromise could be because food allergy is strongly associated with asthma and bronchial reactivity: approximately 50% of food allergic individuals have asthma,^18^, ^19^, ^20^ and many of those without a formal diagnosis of asthma have underlying bronchial hyperreactivity.^20^ Whether food allergic patients without underlying bronchial hyperreactivity and aeroallergen sensitization are less likely to experience respiratory symptoms has not, to our knowledge, been evaluated.

## Clinical Phenotypes in Food Allergy

**Figure 1:**
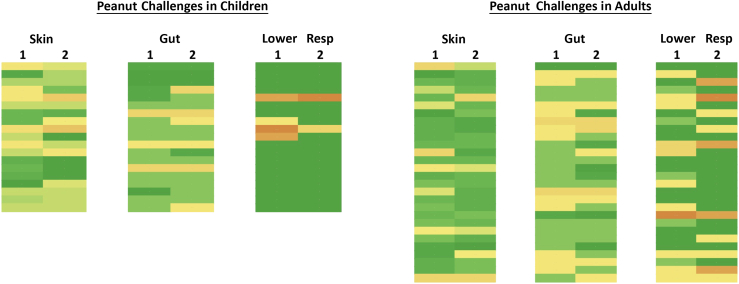
Heat map of symptom severity by organ involvement (skin, gut, or lower respiratory tract) in 19 children and 28 adults undergoing a peanut double-blind, placebo-controlled food challenge on 2 separate occasions (labeled 1 and 2).

There are now a limited number of published studies in which food allergic patients have undergone repeated food challenge, usually to assess changes in eliciting dose (amount of allergen needed to trigger an objective reaction) over time.^21^^,^^22^ We have extensively characterized peanut allergic individuals (n = 57 adults and 64 children), all of whom underwent double-blind, placebo-controlled food challenge, a proportion of whom subsequently underwent a second food challenge months later. We observed a similar pattern of symptoms and organ involvement during the 2 challenges in most individuals (Fig 1). These data are consistent with our clinical observations: many participants report stereotypical reactions; for example, some always experience abdominal pain and nausea, and others do not experience gut symptoms but present with lower respiratory tract involvement; the former might not constitute anaphylaxis, whereas the latter would, although exposure dose is a clear confounder, as explained below.

**Figure 2:**
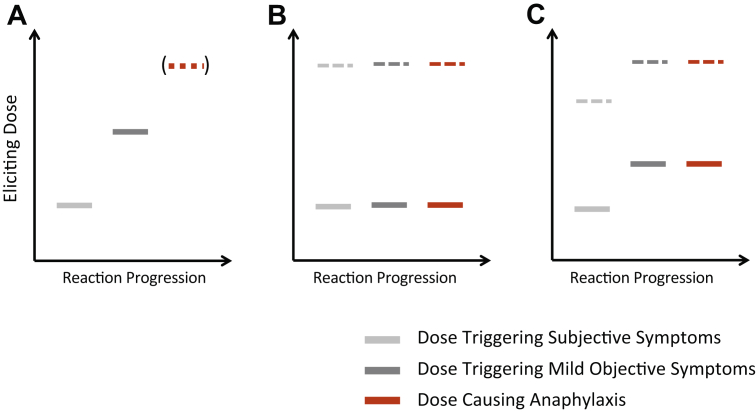
Different patterns of clinical reactivity are seen at food challenge. Many individuals will initially experience subjective symptoms, with objective symptoms appearing with further doses (A). Anaphylaxis will only develop if the food challenge continues. Others will experience anaphylaxis as their first objective symptom: at a dose of allergen exposure with no preceding subjective symptoms (B) or with prior subjective symptoms (C). Note that anaphylaxis can occur at all levels of exposure (both at low levels of allergen exposure, represented by the solid bars, and higher doses, indicated by dotted lines). Reproduced with permission from Turner and Wainstein^23^

These findings may reflect intraindividual and interindividual variability in reaction thresholds, with different patients needing to consume different amounts of allergen to develop specific symptoms on any given reaction occasion.^23^ Furthermore, a range of factors potentially interact with both sensitivity of an individual to a given allergen on any given day as well as severity.^24^ Many individuals will experience initially subjective symptoms, with objective symptoms appearing only with the administration of further doses (Fig 2A). In some individuals, further dosing will cause anaphylaxis, whereas in others, the threshold for any objective symptom and anaphylaxis is similar, and these patients present with anaphylaxis as their first objective symptom (Fig 2B).

We recently undertook unbiased, data-driven analyses to identify differences in the ability of patient serum samples (from peanut allergic individuals) to elicit responses in the mast cell activation test.^25^ Interestingly, using this approach, 5 different clusters of patient phenotypes were identified characterized by different mast cell activation test responses. The differences between the different patient groups were linked to both a patient’s threshold of reactivity (ie, sensitivity or dose needed to trigger objective symptoms) and the resulting severity of those symptoms. This finding raises the possibility of undertaking unbiased, machine-based learning analyses to better understand the existence of different clinical phenotypes in food allergy and whether laboratory tests might be helpful in determining these and potentially identifying patients at greater risk of severe reactions.

## Severe Food Allergic Phenotypes

There is a consensus that severity of reactions cannot be reliably predicted in food allergy^24^; however, predicting the occurrence of anaphylaxis (most of which is not truly life-threatening) and life-threatening anaphylaxis (eg, reactions that are refractory to initial treatment with epinephrine) are different concepts. Prior anaphylaxis appears to be the best predictor of future anaphylaxis (of *any* severity) in studies,^24^ although this finding is confounded by the fact that the occurrence of anaphylaxis is, of course, dependent on sufficient exposure. In a unique study, Wainstein et al^26^ found that up to 75% of peanut allergic children will experience anaphylaxis with sufficient peanut ingestion but most experienced dose-limiting symptoms first. Thus, the absence of a history of anaphylaxis in patients is more likely due to insufficient allergen exposure at previous reactions rather than an inability to have anaphylaxis.

Patients with a history of prior severe anaphylaxis (ie, refractory to initial first-line treatment with ≥2 doses of epinephrine) may be a separate phenotype, but this is a group who to date have not been well studied. In the UK Fatal Anaphylaxis Registry, most cases did not have a prior history of anaphylaxis.^27^ Pouessel et al^28^ followed up 39 children admitted to the intensive care unit (ICU) for food-related anaphylaxis.^28^ Of these patients, 30 (77%) experienced at least 1 further food-related reaction during the follow-up period, 27 of them to the same allergen as the reaction that required the ICU admission. At least 10 children (26%) experienced anaphylaxis, and half required a further ICU admission for management. Although these data are limited, they imply that some patients may have a more allergic phenotype that places them at greater risk of refractory anaphylaxis.

## Why Do Some Patients React With a More Severe Phenotype?

**Figure 3:**
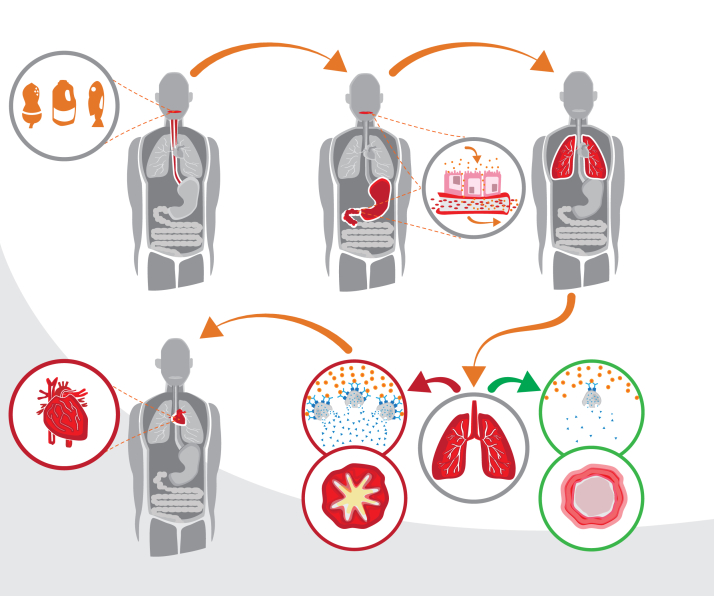
Proposed mechanism for severe IgE-mediated food allergy. Food is initially absorbed in the mouth and across the gastrointestinal tract. The absorbed allergen passes into the bloodstream, where it quickly reaches the respiratory system; those participants who have a higher mast cell density in the lungs have more severe respiratory compromise. Mediators released in the lungs rapidly reach the heart through pulmonary venous circulation and responsible for the cardiac response during IgE-mediated food allergy.

We propose a mechanism for more severe food allergic reactions in Figure 3. In some individuals, systemic absorption of the food allergen (across the oral mucosa^17^ or via the gastrointestinal tract) can occur to a greater degree and more quickly. This process results in rapid distribution through the blood stream, including to the airways. Some individuals, for example, those with underlying sensitization to aeroallergen, may have increased mast cell density in the airways^29^^,^^30^ and could therefore experience more severe respiratory compromise. Release of mast cell mediators in the respiratory tract could lead to bronchospasm, mucous plugging, and secondary cardiac compromise when these inflammatory mediators reach the heart via the pulmonary circulation. In other individuals, perhaps with lower airway mast cell density, mediator release is lower; therefore, these individuals might be at lower risk of severe systemic reactions with lower respiratory or cardiac involvement. Alternatively, individuals at greater risk of severe reactions (only a few patients) may have impaired switch-off mechanisms, such as an inability to compensate for the allergic trigger. Some evidence of this has been reported with respect to the metabolism of the platelet-activating factor.^31^ Thus, severity could result from a combination of not only initial insult but also an inability to compensate once the reaction has been initiated.

## Effect of AIT on Clinical Phenotype

The increase in data from trials of food allergen immunotherapy (AIT) has provided some insight into the effect of AIT on the phenotype of food allergic individuals. AIT, and specifically oral immunotherapy (OIT), is effective in raising the threshold of reactivity (eliciting dose) to the specific food allergen while patients are undergoing treatment.^32^ Interestingly, both the PALISADE (Peanut Allergy Oral Immunotherapy *Study* of AR101 for Desensitization in Children and Adults)^33^ and ARTEMIS (AR101 Trial in Europe Measuring Oral Immunotherapy Success in Peanut Allergic Children)^34^ phase 3 randomized controlled trials, using Aimmune’s AR101 formula for peanut OIT, have found a reduction in severity of reactions and the use of rescue epinephrine at exit food challenge. For example, in PALISADE, 10% of challenged patients in the active group were given rescue epinephrine compared with 53% in the placebo group, despite a similar rate (36% and 37%, respectively) in both groups at baseline challenge.^33^ These data are not entirely surprising in that by raising the reaction threshold fewer reactions occur at exit challenge and, consistent with Figure 2, increasing a patient’s threshold is likely to also increase the threshold for experiencing anaphylaxis.

Chu et al^35^ recently published a meta-analysis of AIT that included 1041 participants across 12 randomized controlled trials. Although AIT induces desensitization, patients also experience an increased rate of allergic reactions, including anaphylaxis, when undergoing treatment. For peanut OIT, the risk of anaphylaxis increases 3-fold, with a relative risk of 3.12 (95% CI, 1.76-5.55). In PALISADE, patients receiving the top dose of active peanut OIT, but not yet established with maintenance therapy, were 5 times as likely to have systemic allergic reactions (8.7% vs 1.7%) as those taking placebo.^33^ As we have previously highlighted, there is a paradox: despite this increased risk, there is clearly demand for OIT from patients and their families.^36^ Many patients and families are clearly willing to tolerate a higher rate of reactions while undergoing OIT; one factor may be that when these reactions occur they happen in a more predictable and controlled way, often with a reduction in severity of symptoms. There may also be other quality of life, psychological, or emotional benefits to OIT, although these benefits remain to be demonstrated. Clearly, we need to understand the effect of AIT on reaction severity and clinical phenotype to better inform patients and families of the risks of AIT and allow them to make a fully informed choice.

Finally, a significant proportion of patients (typically approximately 20%) do not tolerate current OIT regimens. These patients tend to have specific characteristics, for example, higher levels of IgE sensitization and a history of anaphylaxis to the causative allergen, although these features do not currently allow us to predict outcomes in OIT.^37^ The available data suggest a specific patient phenotype that may be prone to more severe and persistent food allergy, which also affects poorer outcomes during OIT. Ironically, these patients arguably have the most to gain from desensitization. Further work is needed to evaluate different protocols, perhaps involving different routes of AIT used in combination or in conjunction with adjunct therapies, such as anti-IgE immunotherapy.

## Conclusion

Data are emerging to confirm our clinical experience that many food allergic patients experience stereotypical symptoms after allergen exposure, both in the community and at supervised oral food challenge, in a manner that varies among patients. However, the existing published data do not yet provide a sufficient evidence base to guide management decisions that would affect patient care. Integrating data sets from different cohorts and applying unbiased machine-based learning analyses may demonstrate specific food allergy endotypes in a similar way to asthma.^38^ Whether this results in improvements in patient management (eg, through facilitating risk stratification or affecting the decision to prescribe an epinephrine autoinjector and, perhaps, the number of devices) remains to be determined, but given our current inability to predict which patients are most at risk of severe food allergic reactions,^24^ this will clearly be an important area of research in the future.
